# Extraction and Research of Crop Feature Points Based on Computer Vision

**DOI:** 10.3390/s19112553

**Published:** 2019-06-04

**Authors:** Jingwen Cui, Jianping Zhang, Guiling Sun, Bowen Zheng

**Affiliations:** 1 School of Electronic Information and Optical Engineering, Nankai University, Tianjin 300350, China; sungl@nankai.edu.cn (G.S.); zhengbwen@mail.nankai.edu.cn (B.Z.); 2 Electrical Engineering and Computer Science, Northwestern University, IL 60208, USA

**Keywords:** computer vision, Kinect v2 sensor, YOLOv3, feature point, visual positioning, point cloud image

## Abstract

Based on computer vision technology, this paper proposes a method for identifying and locating crops in order to successfully capture crops in the process of automatic crop picking. This method innovatively combines the YOLOv3 algorithm under the DarkNet framework with the point cloud image coordinate matching method, and can achieve the goal of this paper very well. Firstly, RGB (RGB is the color representing the three channels of red, green and blue) images and depth images are obtained by using the Kinect v2 depth camera. Secondly, the YOLOv3 algorithm is used to identify the various types of target crops in the RGB images, and the feature points of the target crops are determined. Finally, the 3D coordinates of the feature points are displayed on the point cloud images. Compared with other methods, this method of crop identification has high accuracy and small positioning error, which lays a good foundation for the subsequent harvesting of crops using mechanical arms. In summary, the method used in this paper can be considered effective.

## 1. Introduction

In recent years, with the rapid development of artificial intelligence technology, computer vision, as an important branch of artificial intelligence, has gradually become a hot topic for researchers all over the world. Computer vision is a discipline that studies how to make computers intelligently perceive image data. It is recognized as the most effective and reliable environmental analysis tool [1], which now plays an important role in scientific research and practical applications. Nowadays, the application scope of computer vision has covered various fields such as industry, agriculture, transportation and scientific research. In the agricultural field, computer vision technology has broad application prospects in the identification and picking of crops.

Object recognition is a first problem which we need to consider in computer vision technology. When the camera gets a set of images, it first recognizes the objects in the images. Only after identifying the objects in the images can we partially restore the 3D information of the 2D images. The traditional object recognition methods are generally divided into three stages: region selection, feature extraction, and classifier classification. The traditional object recognition methods have strong dependence on specific images, and the traditional object recognition method has the disadvantages of a complex feature extraction process and many calculations. After a series of improvements and research, Redmon et al. at the University of Washington in the United States in 2016 proposed a convolutional neural network for real-time object detection the YOLO [2] algorithm, which targets objects in the image. The detection speed of this method can reach 155 fps, and the target object can be detected in real time. However, the accuracy of the YOLO algorithm is not very high because of the cancellation of the candidate region [3], but with the continuous improvement of the YOLO algorithm in recent years, the current launch of YOLOv3 has overcome the problem of low recognition accuracy and has improved detection capabilities for small objects. After a series of comparisons with other object recognition algorithms, it was found that the YOLOv3 algorithm has a great number of excellent characteristics in the experiment of this article. Therefore, in this paper, this algorithm was selected to identify crops. The use of this method can greatly improve the recognition accuracy of objects, accelerate the efficiency of crop picking, and create more economic benefits. Therefore, it is quite important to propose this method.

**Figure 1:**
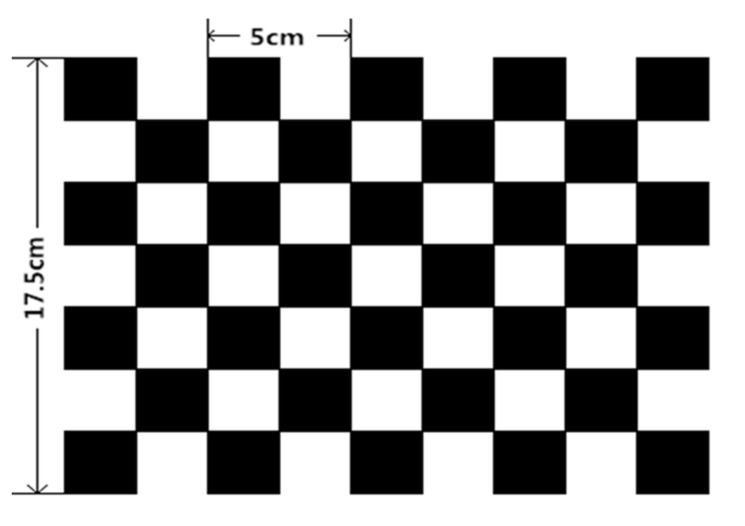
Kinect v2 calibration checkerboard.

In terms of object positioning, both the binocular camera and the Kinect v2 depth camera can determine the position of the object and read its 3D coordinates from the obtained data. The binocular camera can be used both indoors and outdoors, with low interference from light, but it requires complicated configuration and calibration methods. In addition, its depth range and accuracy are limited by binocular baseline and resolution. In contrast, Kinect depth cameras, especially the latest generation of Kinect v2 depth cameras, can overcome these shortcomings. Although the number of sensors is the same as that of the Kinect v1, the principle of the measuring depth is completely different, and the RGB camera installed on the Kinect v2 has a higher resolution than that on the Kinect v1 [4]. At the same time, Kinect v2’s USB interface data transmission rate has also been improved. Because of this excellent performance, the Kinect v2 depth camera introduced by Microsoft was used in this experiment. Before the experiment begins, the Kinect v2 camera needs to be calibrated to obtain the correspondence between the object space coordinates and the pixels in the captured image. The calibration method of the Kinect v2 camera is the Zhang Zhengyou calibration method. This method is simple and effective. It only needs to use a checkerboard printed on white paper. The size of the calibration checkerboard selected in this paper is shown in Figure 1. After the camera is calibrated, the camera’s internal reference matrix, external parameter matrix and distortion coefficient can be obtained.

After the calibration is completed, the Kinect v2 depth camera can obtain accurate RGB images and depth images and then use the YOLOv3 algorithm to identify the target crops in the images. After the recognition is successful, the position of the feature points can be determined. Finally, the official function provided by Kinect v2 is modified to enable it to directly display the 3D coordinates of feature points on the point cloud images. Through the above steps, the two functions of crop identification and feature point coordinate extraction can be completed. In addition, the obtained 3D coordinate information of the target crops can also be published to the ROS topic, and the robotic arm subscribes to the topic to obtain the 3D coordinates of the crops to lay the foundation for the subsequent command of the robotic arm to capture the target crops

**Figure 2:**
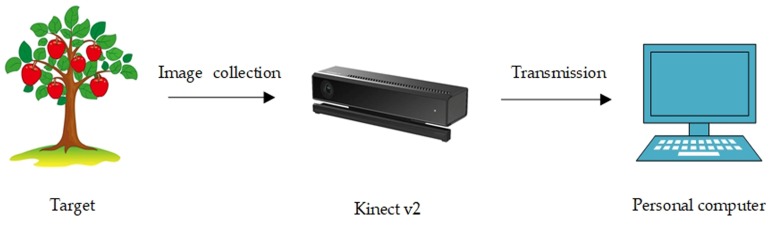
System model.

In practice, computer vision and intelligent robotics technology are often combined. First, the target object must be identified and located, and then the robot must perform the grabbing and navigation operations. The integration of computer vision technology in the field of robots has greatly improved the functions of recognition, positioning, tracking and grabbing, which also reduced the cost and improved the practicality. This test also follows this method to achieve the identification and positioning of the target crops, laying the foundation for the subsequent control of the robotic arm to perform the grab operation. Figure 2 shows the general process framework of the entire experiment. First, the depth camera is used to obtain the crop’s information, and then the information is sent to the computer for further processing and analysis.

## 2. Kinect v2 and Camera Calibration

### 2.1. Kinect v2 Depth Camera

The Kinect depth camera is an XBOX360 somatosensory device developed and promoted by Microsoft in 2010. The purpose is to better capture the depth data of the image. The Kinect camera is a combination of optical- and radar-based systems for human detection, tracking and activity recognition. It consists of a common RGB camera, an infrared camera and a set of microphones [5], which can provide depth signals, RGB images and audio signal streams simultaneously. The RGB camera is responsible for acquiring the color images, the infrared camera acquires the infrared data, and the infrared data obtained by the infrared camera are processed to obtain the depth information of the images. In view of the simple operation, low price and convenient use of the Kinect depth camera, it is widely used in 3D reconstruction [6], object tracking and gesture recognition [7].

**Figure 3:**
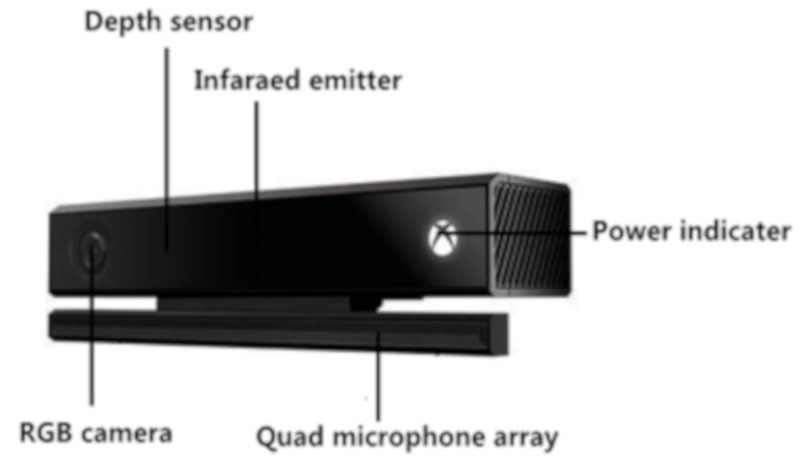
Kinect v2 hardware components.

The devices used for visual positioning in traditional experiments are binocular stereo cameras. This binocular camera is not only inconvenient to use but is also computationally complicated. The Kinect depth camera can completely overcome the above shortcomings. This camera can reliably measure the 3D reachable workspace [8], which is inexpensive and can be easily integrated into practical applications [9] to identify target crops. At present, Microsoft’s Kinect depth camera includes Kinect v1 and Kinect v2. Compared with Kinect v1, Kinect v2’s RGB camera and infrared camera have higher resolution, the USB interface data transmission rate is improved, and the performance is better. Therefore, this experiment uses the Kinect v2 camera, of which the hardware composition is shown in Figure 3.

### 2.2. Calibration Theory of Kinect v2

In the experiment, Kinect v2 acquires the 3D information of the scene through the RGB camera and the infrared camera, and then reads the accurate spatial coordinates of the target crops in the images. In order to achieve this function, Kinect v2 needs to be calibrated so that the RGB images and depth images acquired by Kinect v2 can be matched together. The camera calibration method applied in this paper is the Zhang Zhengyou calibration method.

Zhang Zhengyou’s calibration method, also known as the plane checkerboard calibration method, was proposed in a conference paper published by Dr. Zhang Zhengyou in 1999. This method is easy to implement [10]. During the experiment, only one checkerboard printed on large-size paper is used to complete the camera calibration, and the calibration accuracy of this method is very high. At present, this method has a wide range of applications in the field of computer vision [11,12,13].

First, suppose that there is a point $M{\left[X,Y,Z\right]}^{T}$ in the space, and the coordinates of the projection point of the 2D image plane captured by the camera are $m{\left[u,v\right]}^{T}$. Because Kinect v2 converts 3D points in space into 2D plane points in the captured image, the coordinate system needs to be converted from 3D to 2D. According to the conversion process, the relationship between m and M can be obtained as shown in Equation (1).

(1) $$s\tilde{m}=A\left[R,\;t\right]\tilde{M}$$

In Equation (1), s represents the scale factor of the world coordinate system to the image coordinate system, $\tilde{M}={\left[X,Y,Z,1\right]}^{T}$ and $\tilde{m}={\left[u,v,1\right]}^{T}$ represent the augmented matrices of M and m. $\left[R,\;t\right]$ is an external parameter matrix, where R represents a rotation matrix, t represents a translation vector, and A represents a camera internal reference matrix. The specific expression of A is shown in Equation (2).

(2) $$A=\left[\begin{array}{ccc} \alpha & \gamma & u_{0}\\ 0 & \beta & v_{0}\\ 0 & 0 & 1 \end{array}\right]$$

In Equation (2), $u_{0}$ and $v_{0}$ represent the coordinates of the principal point, and α and β represent the vectors of the u and v axes in the image, which are the differential of the focal length f in the u and v directions. Their expressions are α=f/du and β=f/dv. Since the pixels of the depth camera are not squares of the regular moment, γ is specified here. It is used to indicate the deviation of the scale of the pixel in the u and v directions, that is, the degree of distortion of the two axes. For most standard cameras, the value of γ can be set to zero. However, in the derivation of the algorithm, in order not to lose the generality of the formula, γ is still retained. From the above analysis, Equation (1) can be further recorded as Equation (3). For the convenience of calculation, let $r_{i}={\left[\begin{array}{ccc} r_{1i} & r_{2i} & r_{3i} \end{array}\right]}^{T},\;\left(i\in \left[1,3\right]\right)$.

(3) $$s\left[\begin{array}{c} u\\ v\\ 1 \end{array}\right]=\left[\begin{array}{ccc} \alpha & \gamma & u_{0}\\ 0 & \beta & v_{0}\\ 0 & 0 & 1 \end{array}\right]\cdot \left[\begin{array}{cc} \begin{array}{cc} r_{1} & r_{2} \end{array} & \begin{array}{cc} r_{3} & t \end{array} \end{array}\right]\;\cdot \left[\begin{array}{c} \begin{array}{c} X\\ Y \end{array}\\ \begin{array}{c} Z\\ 1 \end{array} \end{array}\right]$$

In Equation (3), $\left(X,Y,Z\right)$ is the coordinates of the point in the Kinect v2 coordinate system, where X and Y represent the coordinates of a point in the RGB images, and Z is the depth. The depth value of the camera is the same point as the RGB image position. Since the calibration template is a plane, the world coordinate system can be constructed on the plane of Z=0 in the analysis, and then the homography calculation can be performed. Let Z=0, and transform Equation (3) into Equation (4).

(4) $$s\left[\begin{array}{c} u\\ v\\ 1 \end{array}\right]=\left[\begin{array}{ccc} \alpha & \gamma & u_{0}\\ 0 & \beta & v_{0}\\ 0 & 0 & 1 \end{array}\right]\cdot \left[\begin{array}{c} \begin{array}{cccc} r_{1} & r_{2} & r_{3} & t \end{array} \end{array}\right]\cdot \left[\begin{array}{c} X\\ Y\\ 0\\ 1 \end{array}\right]=\left[\begin{array}{ccc} \alpha & \gamma & u_{0}\\ 0 & \beta & v_{0}\\ 0 & 0 & 1 \end{array}\right]\cdot \left[\begin{array}{ccc} r_{1} & r_{2} & t \end{array}\right]\cdot \left[\begin{array}{c} X\\ Y\\ 1 \end{array}\right]$$

Because the change of the Equation (4) is a homography change, let $\;H=A\left[R,\;t\right]$; H can be called a homography matrix. The analysis shows that H is a third-order matrix, one of which is a homogeneous coordinate, so H contains 8 unknowns to be solved. To calculate H, the internal parameter matrix and the external parameter matrix of the depth camera need to be calculated first.

Next, the constraints are used to calculate the parameters in the internal parameter matrix A. From the nature of the rotation matrix, we know that $r_{1}^{T}r_{2}=0$, $\|r_{1}\|=\|r_{2}\|=1$. Let $H=\left[\begin{array}{ccc} h_{1} & h_{2} & h_{3} \end{array}\right]$ and $\left[\begin{array}{ccc} h_{1} & h_{2} & h_{3} \end{array}\right]=\lambda A\left[\begin{array}{ccc} r_{1} & r_{2} & t \end{array}\right]$, where λ is the scale factor. After solving, two constraints in Equation (5) can be obtained.

(5) $$\{\begin{array}{c} h_{1}^{T}A^{-T}A^{-1}h_{2}=0\;\;\;\;\;\;\;\;\;\;\\ h_{1}^{T}A^{-T}A^{-1}h_{1}=h_{2}^{T}A^{-T}A^{-1}h_{2} \end{array}$$

Let $B=A^{-T}A^{-1}$, B is a third-order matrix, and the elements in the matrix are represented by $B_{ij},\;i,j\in \left[1,3\right]$, After calculation, B is found to be a symmetric matrix, so there are only 6 effective elements left. Let these 6 elements form the vector $b={\left[B_{11},B_{12},B_{22},B_{13},B_{23},B_{33}\right]}^{T}$. By calculating the internal parameters of the camera by transforming the constraints, b can be estimated, and then the internal parameters of the camera can be calculated. The result is expressed in Equation (6).

(6) $$\left\{ \begin{array}{c} v_{0}=\left(B_{12}B_{13}-B_{11}B_{23}\right)/\left(B_{11}B_{22}-B_{12}^{2}\right)\\ \lambda =B_{33}-\left[B_{13}^{2}+v_{0}\left(B_{12}B_{13}-B_{11}B_{23}\right)\right]/B_{11}\\ \alpha =\sqrt{\lambda /B_{11}}\\ \beta =\sqrt{\lambda B_{11}/\left(B_{11}B_{22}-B_{12}^{2}\right)}\\ \gamma =-B_{12}\alpha^{2}\beta /\lambda \\ u_{0}=\gamma v_{0}/\alpha -\;B_{13}\alpha^{2}/\lambda \end{array}\right.$$

After calculating the internal parameters of the depth camera, the camera’s internal parameter matrix can be obtained. Then, the outer parameter matrix is solved by the formula $\left[\begin{array}{ccc} h_{1} & h_{2} & h_{3} \end{array}\right]=\lambda A\left[\begin{array}{ccc} r_{1} & r_{2} & t \end{array}\right]$. Equation (7) can be obtained by simplification, where $\lambda =1/\|A^{-1}h_{1}\|=1/\|A^{-1}h_{2}\|$.

(7) $$\{\begin{array}{c} \begin{array}{c} r_{1}=\lambda A^{-1}h_{1}\\ r_{2}=\lambda A^{-1}h_{2} \end{array}\\ \begin{array}{c} r_{3}=r_{1}\times r_{2}\\ t=\lambda A^{-1}h_{3} \end{array} \end{array}$$

After the internal parameter matrix and the external parameter matrix of the camera are obtained, the parameters are optimized by the nonlinear optimization method of maximum likelihood estimation and the radial distortion estimation method. Then, the camera’s distortion factor is obtained to calibrate the depth camera. The above is the principle derivation of Zhang Zhengyou’s calibration. The purpose of this calibration method is to match the RGB images with the depth images. Before calculating the 3D coordinates, the RGB images and the depth images of Kinect v2 are not completely coincident due to problems such as shipping and assembly. Therefore, the camera must be calibrated before calculation. In this way, accurate 3D coordinate data can be obtained in the experiment.

### 2.3. RGB and IR Camera Calibration

The main content of this section describes the calibration process performed by the checkerboard in practical applications, which is implemented by the calibration function provided in OpenCV.

**Step 1:** Calibrate the RGB color camera. 

**Figure 4:**
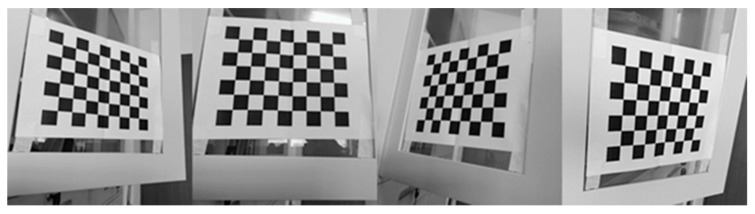
Some RGB image calibration representative results.

Let the RGB camera take about 30 photos from different distances and angles. The reliable line of sight of the Kinect v2 is within 4.5 m [14], so try to keep the distance between the Kinect v2 camera and the target object within this range during the experiment. Figure 4 shows four representative results of RGB image calibration.

**Step2:** Calibrate the depth camera. 

**Figure 5:**
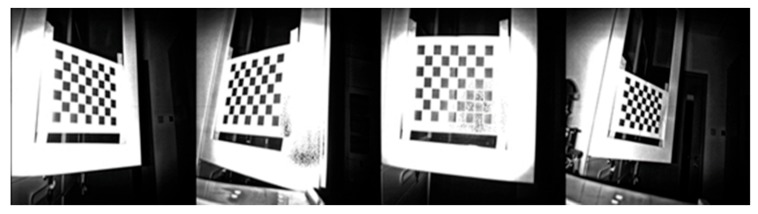
Some depth image calibration representative results.

The specific steps are the same as in step 1, taking about 30 photos from different distances and angles. Figure 5 shows four representative results of depth image calibration.

**Step3:** Calculate the internal parameters. 

The two sets of image sequences obtained in step 1 and step 2 are input into the calibration function of OpenCV for calculation. Then, the internal reference matrix of the RGB camera and the Kinect v2 depth camera can be calculated.

**Step4:** Registration. 

In this process, only the outer parameter matrix of the board relative to the RGB camera and the depth camera in the same scene need to be obtained, and the transformation matrix connecting the two camera coordinate systems can be calculated. According to the experimental results, when the front of the board faces the ray perspective, the matching effect between the RGB image and the depth image calculated by the two camera coordinate system transformation matrix is better.

After performing the above operations, the next step is to test the calibration effect of the camera. The calibration effect can be judged by observing the degree of coincidence between the RGB image and the depth image. If the coincidence degree is high, the calibration can be considered successful. At this time, the Kinect v2 depth camera can establish its own coordinate system and accurately capture the 3D coordinates of any location in the environment. 

## 3. Crop Identification and Feature Point Location

**Figure 6:**
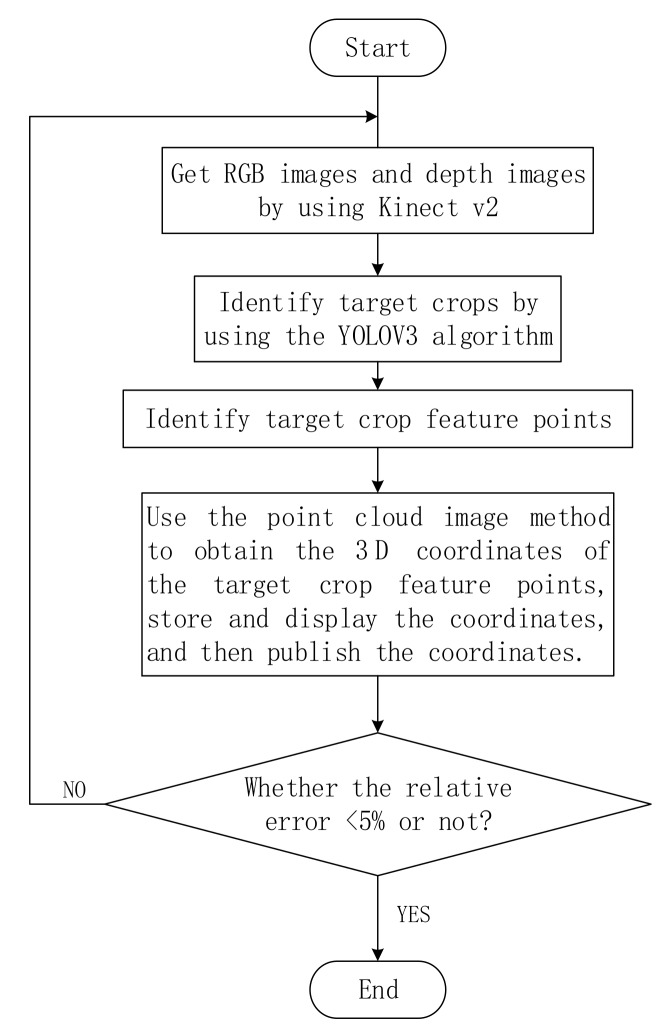
Target crop positioning flow chart.

The flow chart of this paper dealing with the identification and feature point extraction of target crops is shown in Figure 6:

First, the RGB and depth images are acquired using the Kinect camera, and the target crops in the images are identified by the YOLOv3 algorithm; then, the target crop feature points are determined by using the point cloud image coordinate matching method. When the relative error of the obtained feature points is more than 5%, the data are considered to be wrong. It is necessary to return to the first step to re-identify and reposition the target crops.

In the judgment box of the above flow chart, an evaluation criterion for the absolute value of the relative error is established. When the absolute value of the experimental result relative error is greater than the standard, it is necessary to return to the first step to re-identify and locate the target crop. This evaluation standard is an important basis for measuring the quality of this experimental method, so how to determine the absolute value of relative error is very important. When selecting this value, it is based on the “3σ ” principle of normal distribution. When the value of the result falls within the interval $\left(\mu -3\sigma ,\mu +3\sigma \right)$, the probability has reached 99.7%, and it is basically considered that the event will definitely occur. During the course of the experiment, 60 target crop position tests were performed. For the three coordinate axes, we removed the singular values that may be greatly deviated by some random factors, and the relative errors of the remaining p group test results were sorted from absolute from small to large. Let m=⌊p×99.7%⌋, and compare the absolute values of the relative errors obtained by the mth experiment corresponding to the X-axis, the Y-axis, and the Z-axis. Their maximum value is 4.97%, which is 5% after rounding. Therefore, it can be considered that the image recognition and positioning method of this paper can control the absolute value of the relative error within 5%. In other words, the absolute value of the relative error of all the correct results obtained by the experiment will not exceed 5%. For those experiments whose relative error is greater than 5%, those experiments considered to be inaccurate, and these experimental groups need to be re-identified and re-positioned.

### 3.1. Identification of Crops in RGB Images

#### 3.1.1. YOLOv3 Algorithm under the DarkNet Framework

Image processing technology is a technology that converts image information into digital information for computer recognition and related data, and processes image information. It mainly includes image digitization, image enhancement and restoration, image coding compression, image segmentation and image recognition. Traditional image processing methods require manual participation and design [15,16], which is a complex process and requires high demands on the designer’s technology. As people’s demand for image processing grows, the task of image processing is correspondingly required to have high efficiency, high performance and intelligence. In recent years, object recognition algorithms have made great progress. The arrival of the big data era has produced a series of deep learning network structures. These deep learning network structures have shown great advantages in the field of image processing.

Deep Learning is one of the technologies and research areas of machine learning. It implements artificial intelligence in computers by establishing artificial neural networks with hierarchical structure [17]. It has the ability to represent learning [18], which can achieve end-to-end supervised learning and unsupervised learning. The artificial neural network hierarchy complexity of deep learning is called depth. Deep learning uses data to update the parameters in its construction to achieve training goals, and this process is called learning. The first application of deep learning technology in the field of image processing is image recognition. Once applied, the technology has achieved remarkable results. The Alex Net [19] network proposed by Alex et al. is the first deep convolutional neural network applied to image recognition. The subsequent series of developments are based on this.

At present, mainstream object recognition algorithms can be divided into two categories: one is based on Region Proposal’s R-CNN [20,21] algorithm, which is a two-stage algorithm. This method requires the use of a selective search or a Region Proposal generated by the CNN network, and then classification and regression on the Region Proposal. Another type of object recognition algorithm is the one-stage algorithm [22] such as YOLO, SSD, which can directly predict the category and location of different targets using only one CNN network. The second type of YOLO algorithm is used in this paper. The YOLO algorithm is an end-to-end convolutional neural network for common object detection and recognition [23]. 

**Figure 7:**
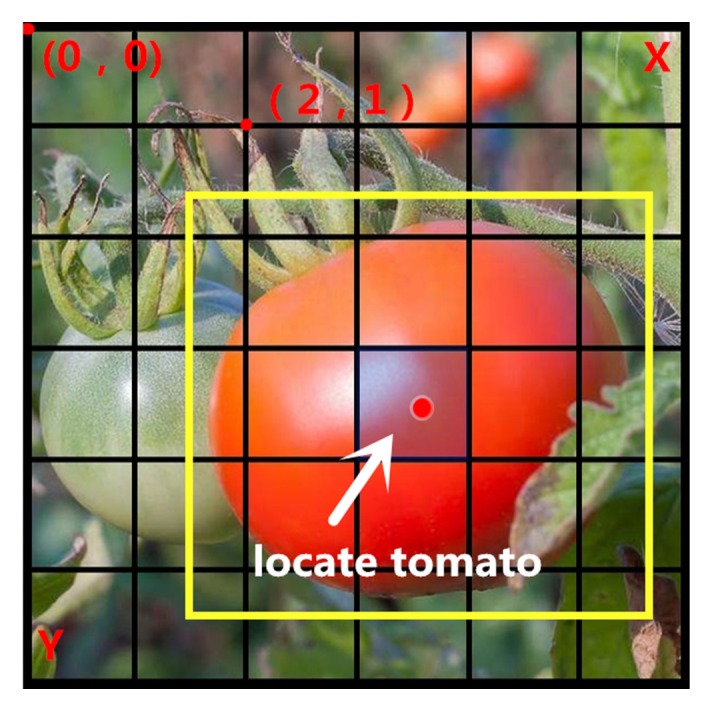
The principle of dividing the grid by the YOLO algorithm.

Common network models include VGG16, GoogleNet and DarkNet [24]. The YOLO algorithm is based on the DarkNet network. Following RCNN, fast-RCNN and faster-RCNN, ROSS Girshick proposes another framework for DL target detection speed. The core idea of this algorithm is to use the whole picture as input, and to detect object based on the global information of the image, and directly return to the position of the bounding box and its category in the output layer. In this algorithm, the input image is segmented into S × S non-coincident grids, each element is used to predict the targets of those center points in the grid, and then the bounding box and the bounding box’s confidence source are predicted. Figure 7 succinctly shows the division principle of the grid in the YOLO algorithm. 

Overall, the YOLO algorithm uses a separate CNN model to achieve end-to-end target detection. In the object recognition process, the YOLO algorithm first changes the input picture size to 448 × 448, and then sends it to the CNN network. Finally, the prediction result of the network is processed to obtain the identified object. Compared to the RCNN algorithm, the YOLO algorithm is a unified framework with faster recognition speed and the end-to-end training process. 

In addition, this algorithm works well for distinguishing between target and background. It can identify the position of multiple targets in one image and display the target name in the image. Because this experiment requires identification of crops grown in a simulated natural environment the crops generally have many in the picture. Using the YOLO algorithm, we can not only accurately identify the crops we need in the complex background of trunks and foliage but also identify the location of multiple crops in the scene. This method is very suitable for this experiment. 

**Figure 8:**
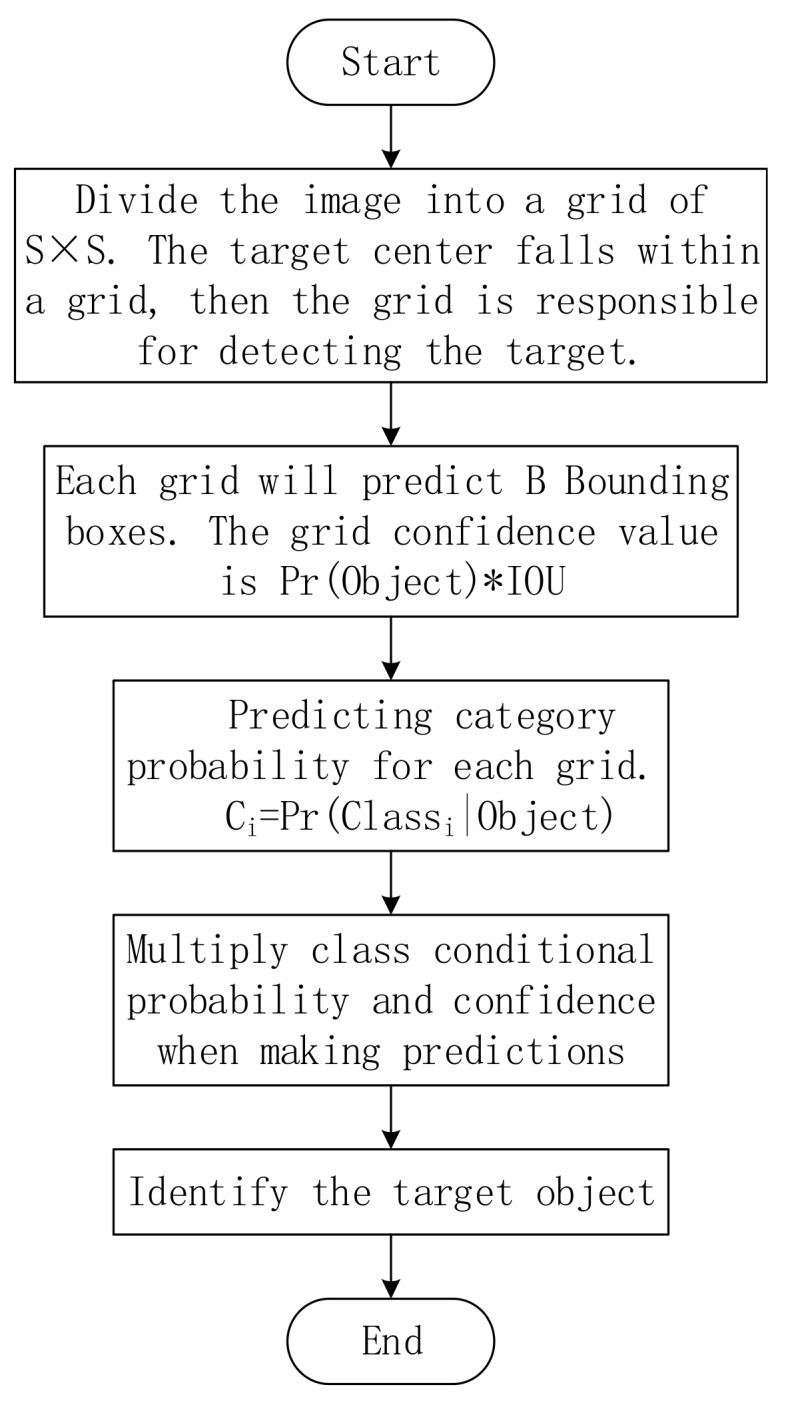
The specific implementation process of the YOLO algorithm.

The flowchart of Figure 8 shows the specific implementation process of the YOLO algorithm.

YOLOv3 is the latest version of the current YOLO algorithm. Compared to YOLOv1 and YOLOv2, YOLOv3 has made great progress in the ability to recognize small objects. It has also significantly improved the ability to detect compact, dense and even overlapping targets. In addition, the training and testing speed of YOLOv3 is also very fast. In this paper, we use the COCO dataset when training the YOLOv3 algorithm. It is a large data set containing image data and annotation information required for multiple tasks such as object detection, key point estimation, semantic segmentation, and image caption. The data set has a wide range of applications in target detection, contextual relationships between targets, and precise positioning of targets in 2D. There are 91 categories in the COCO dataset. Although there are fewer categories than in ImageNet and SUN, there are many images in each class, which is beneficial to locate a particular scene in each class. According to the above analysis, the data set can accurately solve the crop identification problem in this experiment. 

Therefore, for the problem that needs to be solved in this paper, we use the Darknet network model based on the deep learning framework combined with the YOLOv3 algorithm.

#### 3.1.2. Recognition Effect

**Figure 9:**
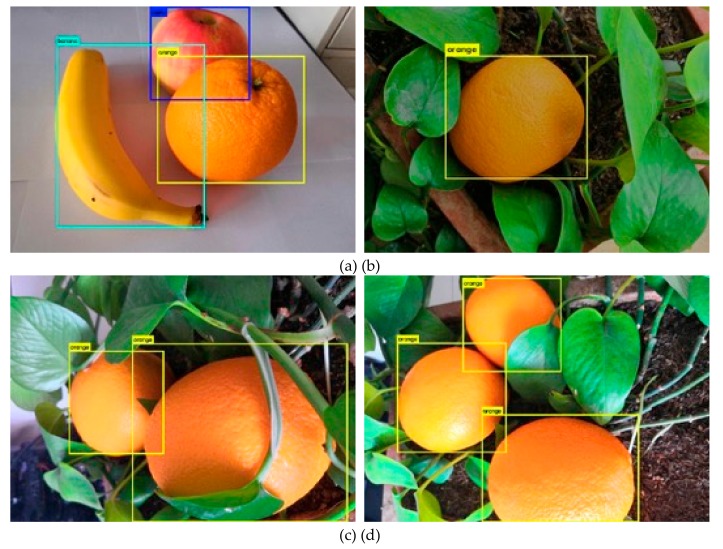
Recognition effect of YOLOv3 under different environmental conditions. (**a**) Identification results under ideal conditions in the laboratory; (**b**) Identification results when the light is dim under a cloudy sky; (**c**) Identification results when the target is blocked by the blade; (**d**) Identification results when the target is occluded by other targets.

In this experiment, the computer’s environment configuration is: Ubuntu16.04+ROS+Kinect v2. Choose different types of crops and use the Kinect v2 camera to take a series of images with the same resolution. Observe the identification of different shapes and colors of crops by the YOLOv3 algorithm. Figure 9 shows the results of image recognition in the experiment. 

Firstly, under the ideal conditions in the laboratory, the YOLOv3 algorithm was applied to the three different shapes of target crops in the scene. The performance of the recognition algorithm is tested by selecting the angle of the target crops to each other, and the recognition result is shown in Figure 9a.

Later, in order to better simulate the characteristics of the target crop in the actual environment, this experiment worked hard to create a simulated environment in the laboratory. Tests were carried out for the occurrence of multiple target crops in the actual scene, poor lighting due to weather, occlusion of the target crops, and mutual occlusion of the target crops. Figure 9b shows the recognition effect when the light is dim under a cloudy sky, and Figure 9c,d respectively show the recognition effects when the plurality of target crops are blocked from each other and blocked by the blades. Through the experimental results, it can be found that even if these interference factors are added in practice, the recognition accuracy of the target crop by the YOLOv3 algorithm is still very high. Therefore, the performance of the image recognition and localization algorithm used in this paper can be considered good.

During the experiment, the image of each identified data folder will be refreshed in the DarkNet folder.

### 3.2. Location and Extraction of Crop Feature Points

#### 3.2.1. Feature Point Definition

The main ideas followed in this experiment are to first identify one or more target crops present in the image using YOLOv3 algorithm introduced in Section 3.1. After the identification is successful, the feature points of the target crop can be determined and the location of the target center point can be extracted. Then, read the 3D coordinates of the target crop and save and display the 3D coordinates. Create a topic and store the acquired data in the topic of Kinect v2. Through this information, we can obtain the spatial location of the target crops, so as to carry out the operation of the next command arm to perform the harvesting of crops. In this process, how to define feature points is also a very critical issue.

The method used to define the feature points in this experiment is to diagonally link the borders identified by the target crops. The line intersection is the center point of the recognition border, where we define the center point of the border as the feature point.

When the target crop is identified using the YOLOv3 algorithm, a rectangular box is created on the RGB graph to mark the target crop, and the name of the target crop is displayed in the upper left corner. Set the four vertices of the rectangle to clockwise from the upper left corner to $A\left(x_{1},y_{1}\right)$, $B\left(x_{2},y_{2}\right)$, $C\left(x_{3},y_{3}\right)$, and $D\left(x_{4},y_{4}\right)$, and set the feature point coordinates to $D\left(x_{4},y_{4}\right)$. The coordinate solution formula of the feature point is as shown in Equation (8).

(8) $$\{\begin{array}{c} x=\frac{x_{2}-x_{1}}{2}\\ y=\frac{y_{1}-y_{4}}{2} \end{array}$$

**Figure 10:**
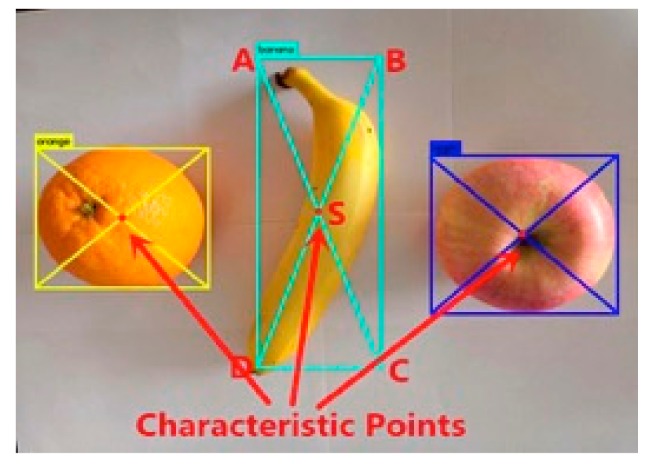
Method for defining characteristic points of crops of different shapes.

In order to better represent the selection method of the feature points, the feature point positions of three different shaped target crops are marked in Figure 10.

In the process of selecting feature points, for the crops with regular shape such as apples, oranges, the feature points obtained by applying the method of this paper are just in the center of them. For such crops, the feature point definition method proposed in this paper is simple and effective. However, in the experiment, the shape of the banana was irregular crescent, so the feature point was not in the center of banana’s vision during the recognition process. In this experiment, after extracting the 3D coordinates of the feature points, the next step is to further control the mechanical arm to capture its spatial position. Since the claw at the end of the robot arm opens a certain angle when the object is grasped, the point can also be regarded as the target crop feature point within the error tolerance. When the feature points indicated in the figure are captured, the implementation of the grab function is not affected. In summary, the definition of this feature point can be feasible in the experiments in this paper.

#### 3.2.2. Feature Point Extraction Method

In the viewer function provided by Kinect v2, by subscribing to the topic released by Kinect v2 under ROS, the RGB color image and depth image acquired by the depth camera can be refreshed in real time, so as to accurately grasp the real-time position of the target crop. After ensuring the real-time nature of the image, the next question to be discussed is how to obtain the target 3D coordinate information. It is also the core issue that needs to be discussed in this session.

Implementing this functionality requires programming in C++. First, create a C++ file in the Kinect_viewer/src directory and then add the ros::Publisher statement to the file initialization. The meaning of this statement is to publish the corresponding topic through ROS. The published message format is geometry_msgs/PointStamped. In addition, the rest of the program content can be modified based on the original viewer.cpp file of Kinect v2. Register the mouse callback function in the cloudViewer function in the original viewer.cpp file, and use OpenCV’s putText function to set the font size and thickness of the 3D coordinates displayed in the image interface. In this experiment, in order to facilitate reading of data, the three-dimensional coordinate color is set to red, the font size is set to 0.5, and the thickness is set to 2.5. Then, remove the extra parameter parsing in the main function to make the program more concise. Finally, add the corresponding build configuration item in CMakeLists.txt under the Kinect_viewer folder. After the C++ program is written, the 3D coordinate function of the point can be read and released by clicking the mouse somewhere. However, for the realization of the automatic reading of the 3D coordinate function, the next step is to use the “button wizard” software. Import the RGB image center point of the target crop into the software to generate a script and finally realize the function of automatic mouse clicking. After completing this step, the extraction of the 3D feature points of the target crop can be achieved.

## 4. Experimental Process and Analysis of Results

**Figure 11:**
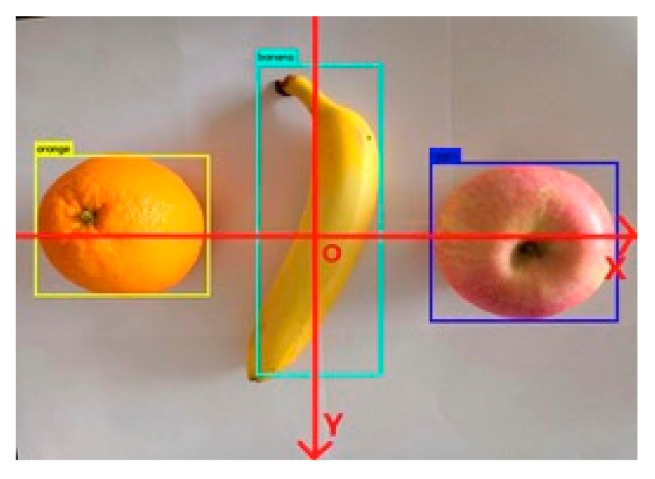
RGB image coordinate system.

Use the terminal input command to start Kinect v2 under Linux system, and then display the acquired color image on the computer screen. In the RGB image, this experiment sets the center of the image as the coordinate origin. Actually selecting other locations, such as the position of the four corners of the image, is also possible. Regardless of where the selected coordinate origin is located, it can be unified by a simple linear transformation. In the actual shooting, due to the background clutter in the laboratory and the unclear display of the three-dimensional coordinates, the clearest part of the captured image will be displayed. If the origin of the coordinate is selected at a certain angle of the picture, the origin of the coordinate cannot be included in the intercepted image to a large extent, and the observation and analysis of the experimental result will have some influence. If the origin of the coordinates is selected in the center of the image, the origin of the coordinates can be placed in the image, which enables more intuitive observation and analysis of the experimental results. In the image, the horizontal direction is the X-axis from left to right, the vertical direction from top to bottom is the Y-axis, and the Z-axis is the depth, and the unit is set to cm, as shown in Figure 11.

**Figure 12:**
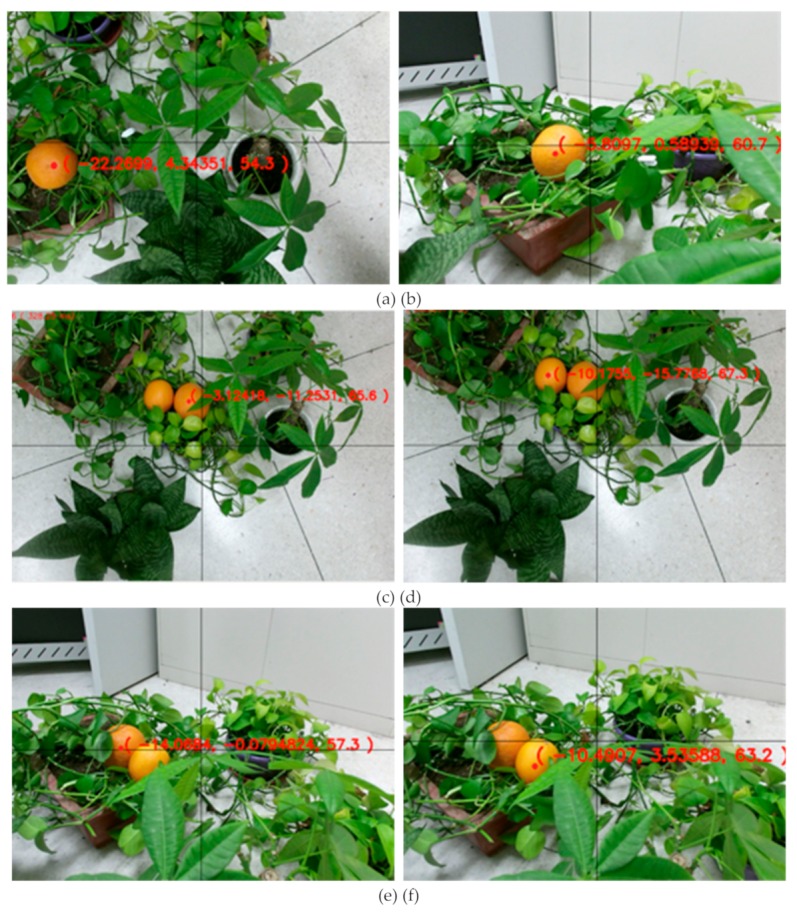
Partial representative results of crop feature point location experiments. (**a**) and (**b**) indicate the feature point location result when there is only one crop; (**c**) and (**d**) indicate the location of the feature points when there are two crops that are not occluded from each other; (**e**) and (**f**) indicate the feature point location results for two partially occluded crops.

In the course of the experiment, efforts were made in the laboratory to create the growth conditions of the target crops in the natural environment. The experiment was divided into three groups. Each group of experiments should take into account the fact that the target crops were blocked by the leaves and uneven light, and each group was subjected to 20 different positioning experiments. The first group of experiments verified that the image contained only one target crop. The second group of experimental images contained two non-occluded target crops. The third group of experimental images contained two target crops that were occluded. In the case of ensuring that the object is at the effective measuring distance of the camera [25,26], try to make the background of each picture clear and concise, which can facilitate the reading of experimental data. Therefore, it is reasonable to crop the pictures that have a cluttered background and where it is not easy to see the 3D coordinate values, and intercept the parts of the figure that contain the coordinates with clear values. Figure 12 is a partial representative result of the experiment. The origin of the coordinates is the intersection of the black X-axis and Y-axis in the figure. The feature points for the target crop are indicated by the red point. The horizontal and vertical coordinate values are obtained by the definition of the YOLOv3 algorithm and the feature points, and the Z value is obtained by the infrared camera. The coordinate value is cm.

Some representative experimental test results are shown in Table 1. The unit of data is cm, and two digits after the decimal point are reserved. 

The average relative errors of the X-axis, the Y-axis, and the Z-axis measured by the multiple experiments performed this time were calculated separately. The expression for solving the average relative error is written in Equation (9).

(9) $$\bar{\sigma }=\frac{\sum_{i-1}^{n}\left|S_{i}\right|}{n}$$

where $\bar{\sigma }$ represents the average value of the relative error, $S_{i}$ represents the relative error value of the i-th experiment of a certain coordinate axis, and n is the number of experiments after removing singular values. The trial was divided into three groups, each of which was measured 20 times.

After calculation, the average absolute values of the average relative error of the X-axis, the Y-axis, and the Z-axis are 2.03%, 2.24% and 0.26%, respectively. By observing the value of the mean absolute error, it can be concluded that the 3D coordinates measured using the method of this paper are relatively accurate.

According to the analysis of experimental data, the method in this paper is accurate in identifying and locating crops. The next step is to determine the evaluation criteria that can be used to measure the accuracy of the algorithm’s recognition and location. This method has been explained in the first paragraph of Section 3. After removing the singular value points, the absolute values of the relative errors of the three coordinate axes in the p experiments are sorted from small to large. Let m=⌊p×99.7%⌋, then take the absolute value of the relative error obtained by the m-th group corresponding to the X-axis, Y-axis, and Z-axis. Taking their maximum value, the maximum value obtained during the experiment was 4.97%, which was recorded as 5% after rounding. From the above analysis, we can conclude that the algorithm for identifying and locating target crops in the environment can control the absolute value of the relative error of the experimental results within 5%. At the same time, in order to better compare with other object recognition and positioning methods, the absolute value of the absolute error of the result is also discussed in this experiment using the same principle. It is concluded that the absolute error obtained in each set of experiments will not exceed 0.5 cm. In addition to the low error, the object recognition and localization algorithm proposed in this paper has the advantages of simple method and stable function. 

In contrast to other methods in the field, a paper published by Dong Jianmin [27] et al. in 2014 proposed a method for automatic recognition and localization of tomatoes based on the Kinect vision system, mainly by using a color image segmentation. In the segmentation result, the Unicom region is analyzed to determine the number of tomatoes and the 2D coordinates of the corresponding pixel points of each tomato. The coordinate values of the three coordinate values obtained by this method and the actual position of the object can be kept below 1 cm, and the positioning algorithm is simple and easy to implement. In 2017, Yang Qiuyi and others from Xihua University used the linear total least squares coordinate transformation method (LTLS) to obtain the coordinates of the feature points of the target object. The error of this method can reach about 0.6 cm, although the error is reduced. The implementation of this method is very complicated.

Because the evaluation indicators used in the above methods are absolute errors, this paper also calculates the average of the absolute values of the absolute errors of the three coordinate axes X, Y and Z calculated before, which are 0.11 cm, 0.48 cm and 0.56 cm, respectively. Combined with the absolute error evaluation criteria obtained above, we compared the absolute errors corresponding to these three methods, and the comparison results are shown in Table 2.

It can be seen from the above analysis that the method for reading 3D coordinates from the point cloud image used in this paper is superior and can meet the needs of this experiment. Only by accurately determining the spatial location of the target crop can the robotic arm determine the correct relative position between itself and the target crop. It is then possible to correctly plan the picking path and perform the picking action [28].

## 5. Conclusions

This paper mainly deals with the RGB image and depth image acquired by the Kinect v2 depth camera and identifies and locates the target crop in the acquired image, and stores and publishes the three-dimensional coordinates. In this experiment, we innovatively combine the YOLOv3 algorithm based on the DarkNet framework with the point cloud image coordinate extraction method. Firstly, the target crops are identified by using the YOLOv3 algorithm, and the feature points of the identified target crops are determined. Finally, the three-dimensional coordinates of the feature points are read by the point cloud image coordinate extraction method. 

After several trials on three different types of crops, it can be found that the three-dimensional coordinates of the final target crops do not exceed 0.5 cm on each axis, and the relative error will not exceed 5%. Compared with other scholars in related fields, the proposed method is not only easy to operate but is also accurate in positioning accuracy. Therefore, it can be considered that the effect of the image recognition and positioning method proposed herein is excellent.

After the appropriate image recognition and localization method is established, the three-dimensional coordinates of the obtained target crop are posted to the topic. The next research direction is how to manipulate the robot arm to automatically move to the 3D coordinates of the target crop and capture the target crop, which is the subject of further research.

## Figures and Tables

**Table 1:** Crop characteristic point location experiment result data.

Group	S/N	Experimental Coordinate Value (cm)			Actual Coordinate Value (cm)			Relative Error (%)		
		x	y	z	x	y	z	x	y	z
Ⅰ	1	−22.26	4.34	54.30	−22.04	4.42	54.20	1.00	−1.81	0.18
	2	−5.81	0.59	60.70	−5.77	0.61	60.30	0.69	−3.28	0.66
	3	3.77	−5.79	55.80	3.71	−5.71	56.20	1.62	1.40	−0.71
	4	0.59	3.46	64.90	0.61	3.35	64.60	−3.28	3.28	0.46
	5	−10.72	7.30	57.30	−10.76	7.38	57.50	−0.37	−1.08	−0.35
Ⅱ	1	−3.12	−11.25	65.60	−2.99	−11.33	66.00	4.35	−0.71	−0.61
	2	3.27	2.45	53.70	3.40	3.18	53.90	−3.82	−22.96 ^1^	−0.37
	3	2.89	4.33	60.60	2.86	4.40	60.80	1.05	−1.59	−0.33
	4	−10.18	−15.78	67.30	−10.29	−15.37	67.10	−1.07	2.67	0.30
	5	4.01	−0.82	53.20	4.04	−0.84	53.40	−0.74	−2.38	−0.37
Ⅲ	1	−14.07	−0.08	57.30	−13.97	−0.08	57.60	0.72	0.00	−0.52
	2	−6.02	4.86	49.10	−6.11	4.90	48.80	−1.47	−0.82	0.61
	3	2.55	3.37	54.90	2.62	3.41	54.70	−2.67	−1.17	0.37
	4	4.66	−2.54	61.60	4.59	−2.65	61.40	1.53	−4.15	0.33
	5	−10.49	3.54	63.20	−10.53	3.48	63.10	−0.38	1.72	0.16

^1^ Singular point, culling this value.

**Table 2:** Comparison of error and complexity of three different methods.

Evaluation Index	Method of This Paper	RGB Image Segmentation	LTLS Coordinate Transformation
Absolute error	<0.5	<1	≈0.6
Complexity	easy	easy	complex
